# Large language models for acute coronary syndrome triage at first medical contact in emergency departments

**DOI:** 10.1038/s41746-026-02904-y

**Published:** 2026-06-17

**Authors:** Xuliang Wang, Wenyao Wang, Ling Li, Jingjia Wang, Xiangyu Yan, Paula Pei Li, Jun Lv, Canqing Yu, Da Liu, Chunli Shao, Jing Chen, Kuo Zhang, Hua Xu, Guan Wang, Mingqi Zheng, Mengdan Miao, Shuanli Xin, Chuanjie Wu, Yuwei Wu, Hongxing Luo, Xiangbin Meng

**Affiliations:** 1 Department of Cardiology and Institute of Vascular Medicine, Peking University Third Hospital, Beijing, China; 2 Institute of Medical Information & Library, Chinese Academy of Medical Sciences and Peking Union Medical College, Beijing, China; 3 School of Disaster and Emergency Medicine, Tianjin University, Tianjin, China; 4 Institute for Hospital Management, Tsinghua University, Beijing, China; 5 Jiahui Medical Research and Education Group, Shanghai, China; 6 Department of Epidemiology and Biostatistics, School of Public Health, Peking University Health Science Center, Beijing, China; 7 Department of Cardiology and Institute of Vascular Medicine, Shenzhen Third People’s Hospital, Shenzhen, China; 8 State Key Laboratory of Cardiovascular Disease, Department of Cardiology, Fuwai Hospital, National Centre for Cardiovascular Diseases, Chinese Academy of Medical Sciences and Peking Union Medical College, Beijing, China; 9 Division of Emerging Interdisciplinary Areas, The Hong Kong University of Science and Technology, Hong Kong, China; 10 Tsinghua-Berkeley Shenzhen Institute, Shenzhen, China; 11 Department of Cardiology, The First Hospital of Hebei Medical University, Shijiazhuang, China; 12 Department of Cardiology, Handan First Hospital, Handan, China; 13 Department of Neurology, Xuanwu Hospital, Capital Medical University, Beijing, China; 14 The Second Dental Center, Peking University School and Hospital of Stomatology, Beijing, China; 15 Institute for Surgical Research, Oslo University Hospital, Rikshospitalet, Oslo, Norway; 16 Department of Physiology, Cardiovascular Research Institute Maastricht (CARIM), Maastricht University, Maastricht, The Netherlands; 17 Pengcheng Laboratory, Shenzhen, China

**Keywords:** Cardiology, Diseases, Health care, Medical research

## Abstract

Acute coronary syndrome triage at first medical contact begins with patient narratives, while definitive diagnosis often requires waiting for cardiac troponin or electrocardiogram results. We developed TriageMaster-70B, a large language model using only patient narratives and vital signs for the triage. In 16,428 retrospective and 512 prospective cases, it showed high sensitivity, with per-case processing 39% faster than independent retrospective cardiologist review. These findings suggest TriageMaster-70B may support rapid, accurate, and interpretable triage in emergency departments. Clinical trial registration: ClinicalTrials.gov NCT06493175; first posted September 20, 2024.

Chest pain is a common presentation in the emergency department (ED)^1,2^, and 10–15% of these patients are ultimately diagnosed with acute coronary syndrome (ACS). The time-critical nature of ACS care is well recognized, with clinical practice emphasizing rapid reperfusion and the minimization of door-to-balloon time in ST-elevation myocardial infarction. Diagnostic delays that postpone reperfusion are consistently associated with higher mortality in myocardial infarction^3^.

Current risk stratification relies on blood tests such as high-sensitivity troponin assays and bedside examinations such as electrocardiography (ECG)^4,5^. At first medical contact (FMC) in less-resourced settings, including rural township health centers in China, these modalities may be unavailable, and even when available, they usually require waiting intervals for data acquisition and interpretation^6,7^. These constraints can slow decision-making at the bedside.

Automated diagnostic algorithms may assist ACS diagnosis and risk stratification and may accelerate clinical decision-making^8^. However, many state-of-the-art automated techniques depend on machine learning models trained on structured ECG and laboratory data^2,9^, whereas in actual practice, triage typically begins with the patient’s narrative and basic vital signs^10^. This mismatch has encouraged the development of artificial intelligence systems designed to estimate the likelihood of ACS and to provide initial recommendations immediately after patients’ symptom descriptions and the collection of vital signs, relying only on FMC information. The recent emergence of large language models (LLMs) suggests that risk stratification and diagnosis of ACS may be performed directly from patients’ narrative information, as LLMs can process unstructured descriptions and integrate diverse inputs into structured reasoning outputs^11,12^.

In this study, we introduce TriageMaster-70B, a 70-billion-parameter LLM adapted for FMC triage. We evaluated its performance in multicenter retrospective and prospective cohorts of patients presenting to the ED with chest pain.

TriageMaster-70B was initialized from an open-source backbone and trained to emulate stepwise clinical reasoning using supervised chain-of-thought prompts, followed by reinforcement learning with verifiable rewards (RLVR) via group relative policy optimization (GRPO)^13^. The training set comprised 90,000 question-answer pairs derived from the electronic health records of 316,245 patients across four hospitals, among whom 19,291 (6.1%) had ACS (Fig. 1A). To reflect conditions at FMC, inputs were restricted to patient-reported symptoms and vital signs, excluding ECG and biomarker data (Fig. 1B).

**Fig. 1:**
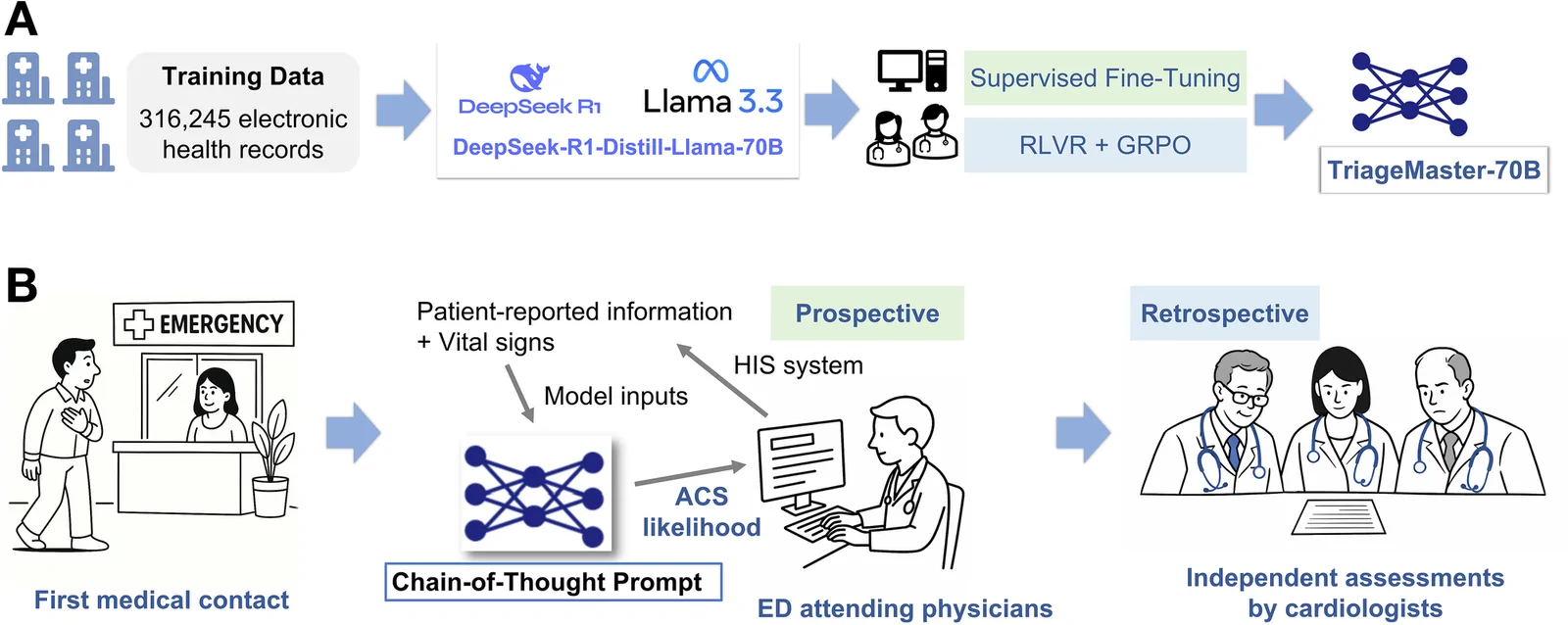
Development and clinical validation workflow of TriageMaster-70B. **A** Model architecture and training pipeline. TriageMaster-70B was initialized from DeepSeek-R1-Distill-LlaMA-70B and fine-tuned using supervised chain-of-thought (CoT) prompts and reinforcement learning with verifiable rewards via group relative policy optimization (RLVR + GRPO), leveraging 316,245 electronic health records from four tertiary hospitals. **B** Clinical deployment and expert verification workflow. Upon first medical contact in the emergency department (ED), patient-reported symptoms and vital signs are entered by ED attending physicians into the hospital information system (HIS). The model uses this information as the input, performs stepwise reasoning via CoT prompts, and generates an acute coronary syndrome (ACS) likelihood score to assist the ED attending physicians with real-time triage. Further, model outputs were independently and retrospectively reviewed by a panel of board-certified cardiologists using only information available at first medical contact. ACS acute coronary syndrome, CoT chain-of-thought, ED emergency department, FMC first medical contact, GRPO group relative policy optimization, RLVR reinforcement learning with verifiable rewards.

The retrospective cohort comprised 16,428 adults (mean age 64.8 ± 13.9 years, 65.4% male) who presented to the ED with chest pain after screening 17,015 cases. Among these patients, 5210 (31.7%) were diagnosed with ACS, including 3991 with non-ST-elevation myocardial infarction or unstable angina and 1219 with ST-elevation myocardial infarction. Hypertension was present in 3863 patients, diabetes in 1295, and a smoking history in 3105, with in-hospital mortality recorded in 445 patients.

For distinguishing ACS from non-ACS, TriageMaster-70B showed higher discrimination than clinician and model comparators (area under the curve (AUC): 0.935, 95% confidence interval (CI): 0.928–0.942), exceeding a ChatGPT-based comparator (AUC: 0.875, 95% CI: 0.869–0.881, *p* < 0.001) and a 20-cardiologist panel (AUC: 0.890, 95% CI: 0.884–0.896, *p* = 0.008). Sensitivity was higher with TriageMaster-70B than with the cardiologist panel (95.3 vs. 91.0%, *p* = 0.01). Diagnostic accuracy was also higher with TriageMaster-70B (90.5 vs. 86.5% for cardiologists, *p* < 0.01) and higher than for ChatGPT (84.9%, *p* < 0.001), Qwen-2 (79.4%, *p* < 0.001), and Lingyi Smart (81.8%, *p* < 0.001). The model further generated stepwise rationales that resembled clinical reasoning and were interpretable to physicians (Fig. 2A). For example, after evaluating symptoms and vital signs, it might state, “The chest pain is pressure-like, radiates to the left arm, and is accompanied by diaphoresis and hypotension, which strongly suggest ACS.”

**Fig. 2:**
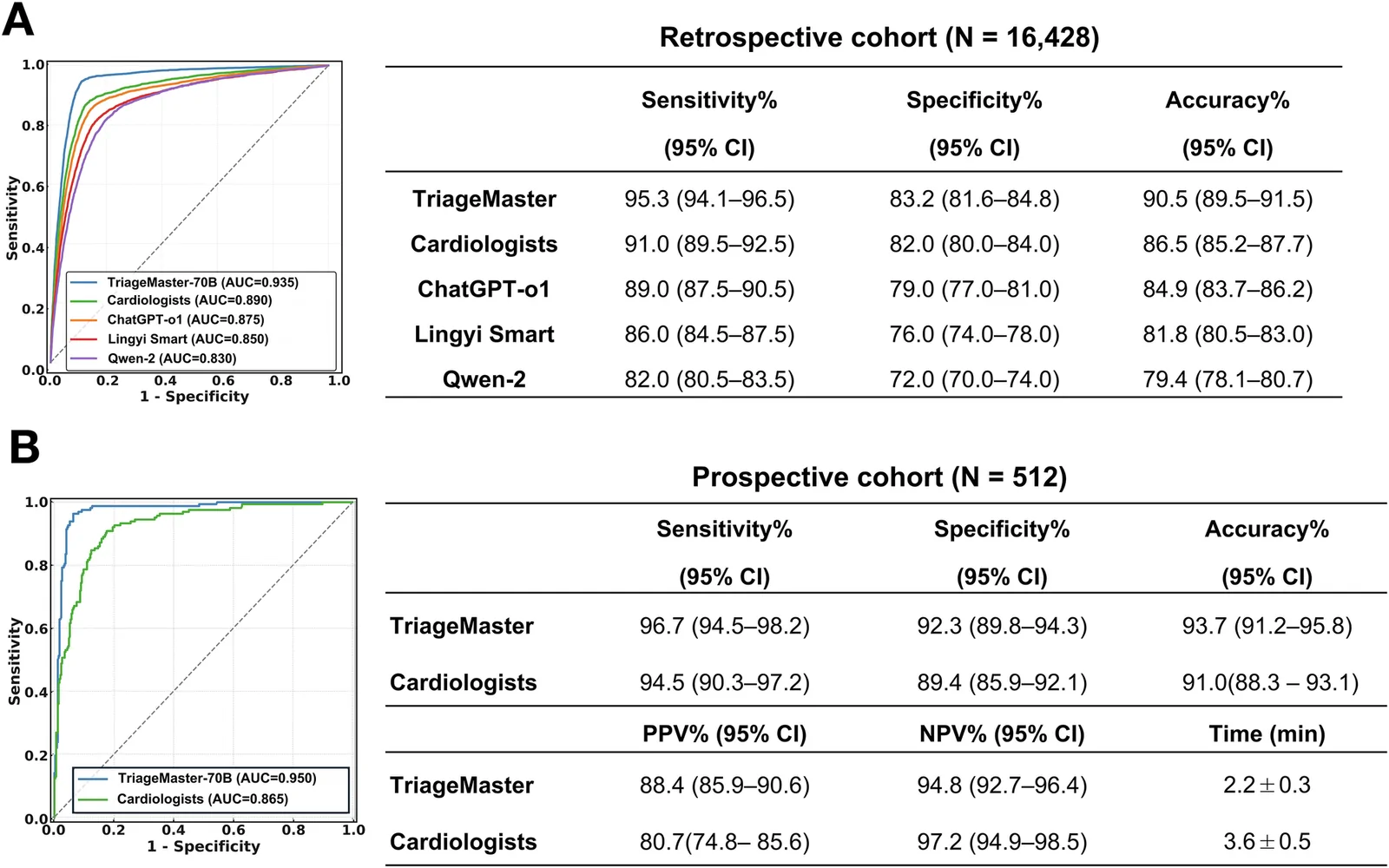
Diagnostic performance of TriageMaster-70B in retrospective and prospective cohorts. **A** Receiver operating characteristic (ROC) curves and diagnostic metrics in the retrospective cohort (*N* = 16,428). TriageMaster-70B outperformed cardiologists and other large language models (ChatGPT-o1, Lingyi Smart, and Qwen-2) across sensitivity, specificity, and accuracy. **B** Results from prospective validation (*N* = 512). TriageMaster-70B demonstrated higher diagnostic accuracy and shorter per-case processing time than independent retrospective review by board-certified cardiologists, with a mean assessment time of 2.2 ± 0.3 vs. 3.6 ± 0.5 min. AUC area under the ROC curve, CI confidence interval, NPV negative predictive value, PPV positive predictive value.

In the prospective study, TriageMaster-70B was integrated into hospital information systems to perform real-time assessment at FMC in EDs while clinicians entered patient-reported information and vital signs (Fig. 1B). The cohort included 512 patients (mean age 65.1 ± 11.9 years, 63.9% male), of whom 164 had ACS. Overall discrimination was high (AUC: 0.950, 95% CI: 0.930–0.970), with sensitivity 96.7% and specificity 92.3%. Among high-risk patients with a Global Registry of Acute Coronary Events score>140, the sensitivity for identifying possible ACS at FMC was 98.0%. Physicians’ final classifications were concordant with the system-generated recommendations in 89% (457/512) of cases, and they rated the TriageMaster-70B interpretability at 4.5 out of 5 points.

To further establish the model performance, all 512 cases were also independently and retrospectively reviewed by a panel of 20 board-certified cardiologists (distinct from the ED physicians who provided real-time care) using only FMC-available information. Per-case processing time was shorter for TriageMaster-70B than for the independent retrospective cardiologist review (2.2 ± 0.3 per case vs. 3.6 ± 0.5 min, *p* < 0.001), a 39% relative reduction. TriageMaster-70B recommended significantly fewer hospital admissions for suspected ACS compared with cardiologists (38.0 vs. 43.2%, *p* = 0.04). Agreement between TriageMaster-70B and cardiologists was high (Cohen’s *κ* = 0.74, *p* < 0.001), and diagnostic accuracy was 93.7% (95% CI: 91.2–95.8) for TriageMaster-70B compared with 91.0% (95% CI: 88.3–93.1) for cardiologists (Fig. 2B).

In this retrospective-prospective study, we developed and validated TriageMaster-70B, an LLM-based system for chest pain triage in EDs. The system integrates into hospital information systems and operates at FMC on patient narratives with vital signs in real time under clinician oversight. Within the fast-paced and information-limited setting of emergency care, it assists clinicians in making earlier and more reliable judgments, which reduces the risk of missed ACS and shortens the time to diagnostic decisions^14^. Stepwise rationales accompany each output to maintain transparency for review and audit^15,16^.

We designed TriageMaster-70B as a binary ACS classifier (ACS-suspected vs. non-ACS) because this mirrors the primary clinical decision at FMC: whether to initiate an urgent ACS workup or pursue alternative diagnoses. While finer risk stratification (e.g., STEMI vs. NSTEMI vs. unstable angina) is clinically relevant, it typically requires ECG and biomarker data unavailable at FMC. The demand for an FMC-first tool arises from the reality that initial decisions regarding suspected ACS are made before definitive test results are available^17^. Clinicians often act before ECG, and high-sensitivity troponin results are available, especially when resources are limited or workflows are congested in scenarios such as EDs^6,18^. Existing automated approaches, including machine learning algorithms, frequently presuppose structured inputs or require manual feature engineering, which constrains their applicability in real time at the start of triage^8,19^. By processing unstructured narratives and vital signs that are available immediately at FMC, TriageMaster-70B produces early probability estimation that helps clinicians identify high-risk ACS cases and prioritize bedside monitoring^20^. This capability allows risk to be estimated at the very start of care, narrowing the critical time gap before confirmatory testing becomes available.

The value of this approach is further supported by comparative evaluation. In head-to-head assessments against experienced cardiologists and general-purpose generative models such as ChatGPT, TriageMaster-70B achieved stronger overall discrimination and higher diagnostic accuracy using FMC information alone^21,22^. The same pattern is held in retrospective benchmarking and during prospective deployment within hospital systems, which indicates that our model performance is not confined to curated datasets. Moreover, physicians’ final classifications frequently agreed with the model recommendations, and physicians rated interpretability positively. TriageMaster-70B’s per-case processing time was also shorter than the time required by cardiologists for independent retrospective review in our prospective validation cohort.

Clinical concordance with model output usually depends on how clearly the model communicates its reasoning process^23^. Utilizing the chain-of-thought procedures, TriageMaster-70B generates explicit stepwise reasoning that parallels clinical thought processes^24^. Physicians can inspect intermediate assumptions, reconcile outputs with bedside findings, and identify situations that warrant escalation or additional examinations. This transparency addresses concerns about black-box recommendations for deep learning models^25^.

Future application extends the FMC-first concept beyond in-hospital settings. Integration with prehospital services and telemedicine can enable earlier assessment during ambulance transport or remote consultation. At home, a privacy-preserving mobile application could allow patients with chest pain to submit narrative information and vital signs for preliminary ACS risk screening^26^. Moreover, TriageMaster-70B incorporation into primary care systems may assist earlier ACS identification and facilitate timely patient referral to higher-level hospitals in resource-limited settings such as rural township health centers in China, which could not only improve patients’ outcomes but also contribute to more equitable access to healthcare resources. The effectiveness and safety of TriageMaster-70B are being evaluated in an ongoing multicenter randomized controlled trial.

This study has several limitations that should be acknowledged. First, the prospective validation was conducted in a single country with all sites in China, and the modest sample size in the prospective validation cohort may restrict our model’s generalizability across healthcare systems with different demographics and resources. Additionally, the ACS prevalence (31.7%) in our retrospective cohort exceeds the typical prevalence of chest pain in community settings (10–15%), reflecting the tertiary referral nature of our study sites. Thus, our model performance should be re-evaluated in external cohorts. Our ongoing multicenter randomized controlled trial and planned international collaborations will help expand this external validation, with the randomized design incorporating treatment-related clinical endpoints. Second, the model was trained and evaluated using only patient narratives and vital signs without ECG or biomarker data. This focus reflects its intended role at FMC, but future versions may integrate additional inputs to improve diagnostic precision. Finally, because the workflow depends on language input, outputs are sensitive to the completeness and phrasing of patient narratives and to variation in data entry across sites. Chain-of-thought explanations support clinician review but may appear persuasive even when derived from incomplete information, underscoring the need for standardized intake templates, input validation, and human-in-the-loop safeguards in emergency settings. Third, although comparator models such as ChatGPT were evaluated using de-identified data transmitted via official APIs, clinical deployment of cloud-based LLMs raises data sovereignty concerns that may limit their applicability in privacy-sensitive environments. TriageMaster-70B addresses this limitation through local, offline deployment within hospital information systems, eliminating external data transmission entirely. While the model training required substantial computational resources (64×NVIDIA H100 GPUs), it is important to emphasize that these requirements apply exclusively to the training phase. For clinical deployment, quantized variants of TriageMaster-70B such as INT4/GGUF formats can operate on commodity hardware, including Apple Silicon workstations (e.g., Mac Studio with M2 Ultra, 192GB unified memory), substantially reducing infrastructure barriers in resource-limited or offline clinical settings. Nevertheless, future work should continue to explore model compression and edge deployment strategies to further broaden accessibility across diverse healthcare environments.

In summary, TriageMaster-70B demonstrates superior diagnostic accuracy for early ACS triage at FMC in EDs, outperforming both cardiologists and LLMs in multicenter cohorts. Its transparent reasoning achieves high concordance with clinicians’ final classifications, underscoring the potential of LLM-based systems to enhance rapid, trustworthy decision-making in high-stakes acute care settings.

## Methods

### Study design and participants

We conducted a retrospective cohort study and a prospective pilot deployment across four tertiary hospitals in China: Peking University Third Hospital (Beijing), First Hospital of Hebei Medical University (Shijiazhuang), Handan First Hospital (Hebei), and Xuanwu Hospital of Capital Medical University (Beijing). The retrospective cohort included adults (≥18 years) presenting to the ED with chest pain between June 2021 and June 2024. Cases with incomplete records (defined as missing chief complaint or ≥2 vital sign parameters) were excluded according to a prespecified protocol, yielding an exclusion rate of 3.4% (587 of 17,015 screened cases). The prospective pilot enrolled consecutive adult chest-pain presentations from October to December 2024 at the same sites. The prospective study was registered on ClinicalTrials.gov (NCT06493175; first posted September 20, 2024). All participants in the prospective study provided written informed consent, and the protocol was approved by the Peking University Third Hospital Ethics Committee (Approval #M2023828). In both cohorts, only information available at the time of FMC was used for model input, including the chief complaint, history of present illness, past medical history, and vital signs. Notably, ECG and cardiac biomarker results were excluded to simulate the initial triage scenario before definitive testing. The final diagnosis of ACS, including ST-elevation myocardial infarction, non-ST-elevation myocardial infarction, and unstable angina, was established by cardiologists according to the 2025 ACC guideline-defined criteria, incorporating ECG changes and troponin results where appropriate^10^.

### Model development

TriageMaster-70B was initialized from the open-source DeepSeek-R1-Distill-Llama-70B model, a 70-billion-parameter model produced by distilling the reasoning capabilities of DeepSeek-R1 into the Llama-3.3-70B architecture. We fine-tuned this model on a large corpus of ED clinical interactions to specialize it for chest pain triage. The training dataset comprised 316,245 de-identified patient-clinician dialog transcripts from the four participating hospitals. From these, approximately 90,000 question-answer pairs were derived for supervised fine-tuning. Each dialog described the patient’s symptoms, medical history, and initial examination findings. To mirror real triage discussions, these dialogs were restricted to patient-reported information and vital signs, with ECG and laboratory data intentionally excluded. Model training was conducted in two stages. In the first stage, we performed supervised fine-tuning with chain-of-thought prompts to teach the model a stepwise clinical reasoning process. These prompts guided the model to decompose the triage decision into logical steps, such as “First assess the character and severity of chest pain, then consider risk factors and vital signs, next evaluate for features suggestive of ACS, and finally provide the likelihood of ACS.” By using structured reasoning prompts inspired by the chain-of-thought technique, the model was trained to generate transparent intermediate reasoning before providing a final diagnosis. In the second stage, we applied RLVR to refine the model’s decision-making. Specifically, we employed the GRPO algorithm, a variant of proximal policy optimization that estimates baselines from group-sampled outputs rather than a separate critic model. GRPO rewards the model for logically consistent reasoning chains and penalizes diagnostic errors by comparing sampled outputs against verified clinical labels. This RLVR phase leveraged automated verification of clinical accuracy alongside human expert assessments of reasoning quality to optimize the model outputs. Model training consumed approximately 18,000 GPU hours on a 64×NVIDIA H100 cluster, utilizing mixed-precision computation and a maximum sequence length of 6144 tokens to accommodate detailed dialogs. Hyperparameters such as a learning rate of 3 × 10^−6^ (cosine decay) and batch size equivalent to 1024 (with gradient accumulation) were used to ensure stable learning. The final TriageMaster-70B model produces two outputs for each case: (1) a binary prediction of whether the patient is having an ACS, and (2) a written chain-of-thought explanation outlining the reasoning behind that prediction.

### Evaluation and triage workflow

In the retrospective analysis, we evaluated TriageMaster-70B’s diagnostic performance against three state-of-the-art generative LLM-based models and a panel of human experts. The comparator LLMs were ChatGPT (OpenAI, version October 2023), Qwen-2 (Alibaba Group’s 13B-parameter general LLM), and Lingyi Smart (a Chinese medical LLM, version 2.2.0)^27^. For a fair comparison, each model received identical structured prompts containing the same case information in its native language (Chinese for Qwen-2 and Lingyi Smart, while English for ChatGPT, with professional medical translation verified by bilingual physicians). All prompts instructed the model to determine ACS likelihood and provide reasoning. ChatGPT evaluations were performed through the official API with all patient data de-identified prior to transmission to comply with data protection regulations. We structured the prompts in a standardized chain-of-thought format for all models to encourage a reasoning process, though only TriageMaster-70B was explicitly trained to produce the reasoning steps. The human expert panel consisted of 20 board-certified cardiologists (average 12 years of clinical experience) who independently reviewed each case’s initial presentation. For every case, each cardiologist provided an independent binary classification (ACS-suspected vs. non-ACS). Then the panel-level continuous score for that case was defined as the proportion of the 20 cardiologists who classified it as ACS-suspected (range 0–1.0). The cardiologist-panel ROC curve and the panel-level AUC reported in this study were derived from these per-case panel-level continuous scores, with 95% CIs obtained via bootstrap resampling (1000 iterations). Panel-level sensitivity, specificity, accuracy, and F1-score were calculated from the corresponding majority-vote binary classification (ACS-suspected when ≥11 of 20 cardiologists rated the case as such). The same metrics, including AUC with bootstrap 95% CIs, were calculated for each LLM comparator using its model-generated probability outputs. To compare diagnostic performance, we used DeLong’s test for pairwise AUC comparisons on correlated receiver operating characteristic curves, McNemar’s test for paired comparisons of sensitivity and specificity, and chi-square tests for proportions. Model calibration was assessed using the Hosmer–Lemeshow goodness-of-fit test and visual calibration plots, and decision thresholds were determined by maximizing the Youden index on validation data. All tests were two-sided, and *p* < 0.05 was considered statistically significant.

In the prospective pilot deployment, TriageMaster-70B was integrated into the ED triage workflow using a custom interface. Upon a patient’s arrival and initial nursing assessment, the relevant clinical details (symptoms and history as described by the patient, plus vital signs) were entered into the system. The model then automatically generated a real-time triage recommendation, consisting of an ACS risk estimate (percentage) with an explanatory chain-of-thought narrative (Fig. 1B). ED physicians on duty (attending-level emergency physicians, not cardiologists) were blinded to the retrospective study results and were briefed only on the model’s general purpose without exposure to prior performance data. Physicians first documented their independent clinical impression before viewing the model output, after which they had discretion to incorporate or override the model recommendation. For each case, we recorded whether the physician’s final triage disposition was concordant with the model recommendation (defined as agreement on the binary ACS-suspected vs. non-ACS classification), as well as the triage decision time (defined as the interval from completion of initial data entry to the physician’s documented triage disposition, measured via electronic timestamp logs). After evaluating each patient, physicians rated the interpretability of the model output on a 5-point Likert scale (1 = not understandable, 5 = very clear reasoning). The primary outcomes in the prospective study were the model sensitivity and specificity for identifying ACS (using the final hospital diagnosis as ground truth), the rate of concordance between physicians’ final classifications and the model recommendations, and the average triage processing time with vs. without the model. We also measured the concordance between model-predicted diagnoses and physician diagnoses using Cohen’s kappa. All analysis was performed with Python/R statistical packages.

## Data Availability

De-identified patient-level data are available upon reasonable request to the corresponding author, subject to institutional approval.

## Code availability

The TriageMaster-70B fine-tuning pipeline is accessible upon request through academic collaboration agreements. The model and its code are publicly available on Hugging Face at https://huggingface.co/mengxiangbin/clinic-research-TriageMaster-70B. To ensure integrity and prevent data leakage, the training and evaluation datasets were strictly separated. All splits were performed at the patient level (no patient appeared in more than one split), temporal ordering was enforced so that training data preceded evaluation data chronologically, and hospital-level stratification ensured representation across sites. All input fields were programmatically restricted to chief complaint, history of present illness, past medical history, and vital signs. Any record containing ECG or cardiac biomarker values in the input was automatically excluded via rule-based filtering before model inference.
